# Artificial intelligence application in the prediction of spontaneous preterm birth by cervical length in the first trimester of pregnancy: Comparison of three measurement methods

**DOI:** 10.1002/ijgo.70744

**Published:** 2026-01-09

**Authors:** Yi‐yun Tai, Bor‐Yann Tseng, Zhu‐Han Yang, Chi‐Hua Yu, Liona C. Poon

**Affiliations:** ^1^ Department of Obstetrics and Gynecology National Taiwan University Hospital Taipei Taiwan; ^2^ Department of Engineering Science National Cheng Kung University Tainan Taiwan; ^3^ Department of Obstetrics and Gynaecology The Chinese University of Hong Kong Hong Kong SAR China

**Keywords:** artificial intelligence, cervical length measurement, ResUNet model, spontaneous preterm birth, transvaginal ultrasound

## Abstract

**Objectives:**

The current study evaluates the efficacy of artificial intelligence (AI)–assisted measurement of cervical length (CL) in predicting spontaneous preterm birth (sPTB), comparing the traditional single‐line and two‐line methods with the innovative AI‐line method in the first trimester of pregnancy.

**Materials and Methods:**

This study is a retrospective secondary analysis of ultrasound images collected prospectively from women with a viable singleton pregnancy who were undergoing Down syndrome screening at Prince of Wales Hospital, Hong Kong SAR. CL was measured using transvaginal ultrasound, with a secondary analysis of archived 1664 images acquired during a prospective study and processed through a ResUNet‐based model. This model, combining UNet and ResNet architectures, a modified ResUNet framework, aimed to overcome the limitations of current measurement techniques by providing a more accurate prediction of CL, particularly in cases where the cervix is curved.

**Results:**

The AI‐line method demonstrated superior accuracy in predicting sPTB at <37 and <32 weeks of gestation compared with conventional methods, with higher areas under the receiver operating characteristic curve (AUROC). The AUROC of CL measured by the AI‐line method (0.676 [95% CI, 0.616–0.735], *P* < 0.05) in predicting sPTB at <37 weeks of gestation was significantly higher than the single‐line (0.537 [95% CI, 0.474–0.6]) and two‐line (0.54 [95% CI, 0.473–0.66]) methods. For the prediction of sPTB at <32 weeks of gestation, the AI‐line method achieved an AUROC of 0.777 (95% CI, 0.703–0.850).

**Conclusion:**

The AI‐line method offers a more accurate measurement of CL in the first trimester, showing potential as a tool for early screening of sPTB risk. The study's results could significantly influence clinical decision‐making, providing a basis for the potential future clinical application of AI in prenatal care.

## 1 INTRODUCTION

Prediction of preterm birth (PTB) is essential for effectively managing the risks of significant perinatal morbidity and mortality rates. Cervical length (CL), particularly as assessed by transvaginal sonography in the second trimester, is a well‐established predictor of spontaneous PTB (sPTB). However, the effectiveness of CL measurement in the first trimester remains controversial, with studies yielding inconsistent results.^1^, ^2^, ^3^, ^4^

One explanation for these discrepancies is that the undeveloped lower uterine segment is often visualized as the isthmus in early pregnancy, leading to potential misinterpretation of the location of the internal os.^5^ In addition, research indicates that the two‐line method, which measures CL as the sum of two lines: one from the internal os to the greatest curvature, and the other from the end point of the first line to the external os, may be more accurate for the typically curved cervix in the first trimester.^6^ Consequently, it is crucial to develop more precise measurement techniques for early pregnancy, especially for curved cervices.

Artificial intelligence (AI), particularly deep‐learning models, can efficiently process large volumes of medical imaging data, reducing manual labor costs and improving efficiency. Models such as Unet are effective in ultrasonographic image segmentation, addressing missing features during extraction, and performing well with limited data sets.^7^, ^8^, ^9^ Furthermore, Resnet models can solve training difficulties associated with increased model depth, enhancing performance in complex image analyses.^10^, ^11^ Deep‐learning algorithms have proven effectiveness in segmenting ultrasound images, improving diagnostic precision and supporting the development of predictive models for CL measurement.^7^, ^12^, ^13^ Our research employs AI, a modified ResUNet framework, to evaluate three methodologies for measuring CL in the first trimester, aiming to enhance predictive accuracy for sPTB. This study aims to contribute to prenatal care by refining predictive models in predicting sPTB in the first trimester of pregnancy.

## 2 MATERIALS AND METHODS

### 2.1 Study population

This study is a retrospective secondary analysis of ultrasound images collected prospectively from women with a viable singleton pregnancy who were attending a screening appointment for Down syndrome at 11 + 0 to 13 + 6 weeks of gestation, from June 2018 to July 2020, at the Prince of Wales Hospital, Hong Kong SAR.^6^, ^14^ We excluded miscarriage, stillbirth, termination, missing outcome data, medically indicated PTB, or poor‐quality/incomplete ultrasound images. Inclusion criteria were singleton pregnancy at 11 to 13 + 6 weeks, available pregnancy outcome, and a good‐quality midsagittal cervical image. Figure S1 illustrates the flow of participants in the study. All eligible women provided written informed consent before participation. In this study, maternal history (including age, weight, height, body mass index, racial origin, smoking habit, method of conception, parity, obstetrical history, previous PTB, and cervical surgery history) and CL were recorded in all cases in the base‐cohort population.

### 2.2 Ultrasound assessment

CL measurements (single‐line and two‐line methods) were performed by six Fetal Medicine Foundation (FMF)–certified sonographers (>5 years' experience) according to the FMF protocol (www.fetalmedicine.org). Operators were blinded to pregnancy outcomes. Image quality and consistency were audited by a senior maternal–fetal medicine specialist. In brief, all women had an empty bladder and were in a modified lithotomy position. The transducer was introduced into the anterior fornix. After having identified the entire length of the endocervical canal in the sagittal plane, the position and pressure of the transducer were adjusted, and the ultrasound image was magnified by ensuring that the cervix occupied 50% to 75% of the image. Care was taken to visualize the endocervical canal clearly without excessive pressure being applied to the cervix to avoid artificial lengthening, and the image was saved and stored.

### 2.3 Study design

**FIGURE 1:**
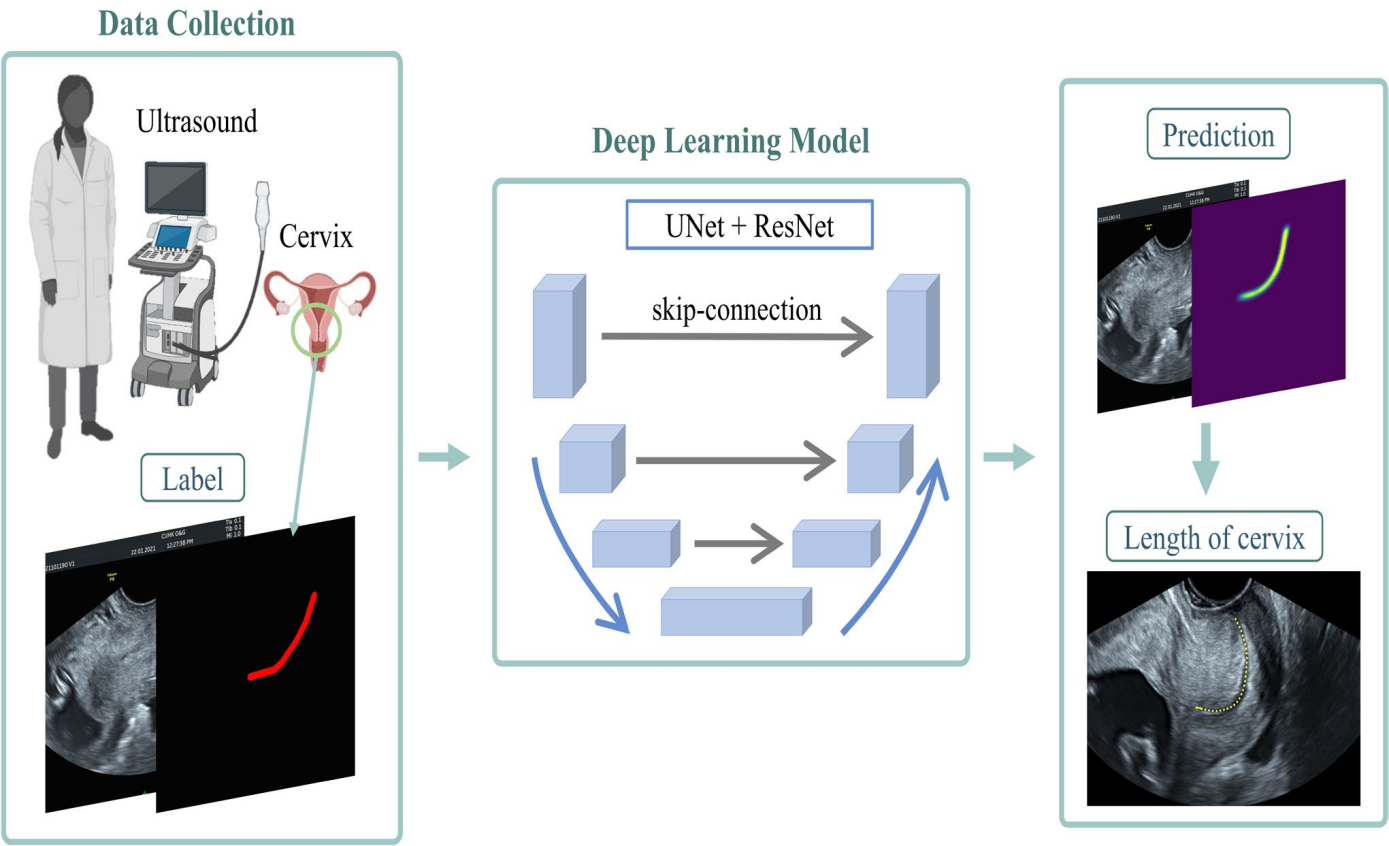
Flowchart of ultrasound image prediction process. We developed a deep‐learning model combined with UNet and ResNet models to analyze ultrasound images. In ultrasound image prediction, we can accurately label the endocervical canal of the cervix in an ultrasound image.

Figure 1 illustrates the three‐stage process of the study. In the data‐collection stage, transvaginal ultrasound images of the cervix were obtained at 11 + 0 to 13 + 6 weeks of gestation from participants enrolled in the first‐trimester screening program. For each image, manual tracings of the endocervical canal (ground truth labels) were delineated using the in‐house annotation software by two independent obstetric sonographers, each with >5 years of experience in maternal–fetal imaging. These tracings were reviewed and confirmed by a senior maternal–fetal medicine specialist to ensure accuracy. Interobserver agreement was assessed using the Dice Similarity Coefficient (0.97 ± 0.01) and SSIM (0.95 ± 0.02), confirming excellent reproducibility.

In the deep‐learning stage, a modified UNet+ResNet architecture with skip connections was used for automated segmentation of the cervical canal from ultrasound images. The data set was divided into training (75%), validation (15%), and testing (10%) subsets. Finally, in the prediction stage, the model generated a segmented curve representing the cervical canal, from which the CL was automatically measured against and compared with manual ground truth tracings for validation.

### 2.4 Data set

In our data set, all ultrasound images were analyzed. Among these, 75% of the images were allocated to the training data set, 15% to the validation data set, and the remaining 10% to the testing data set. The division of the data was based on the methodology suggested in a research study, where the ratio of training to testing data was proposed as 90% to 10%.^11^ We adapted this approach by allocating a portion of our training set to create a validation set. This allows for more detailed monitoring of the model's performance during training, enabling adjustments to be made more effectively to optimize learning outcomes. We resized the images to a 512 × 256 aspect ratio to match the model's input requirements.

To enhance the model's performance, we employed image‐flipping techniques. We effectively augmented the available information for our deep‐learning model by generating multiple versions of our images with different orientations. This approach allowed us to provide additional learning opportunities without the need for labor‐intensive tasks such as gathering and labeling more training data.

### 2.5 ResUnet architecture

We developed a deep‐learning model combined with UNet and ResNet models to analyze ultrasound images. In ultrasound image prediction, we can accurately label the endocervical canal of the cervix in an ultrasound image.

**FIGURE 2:**
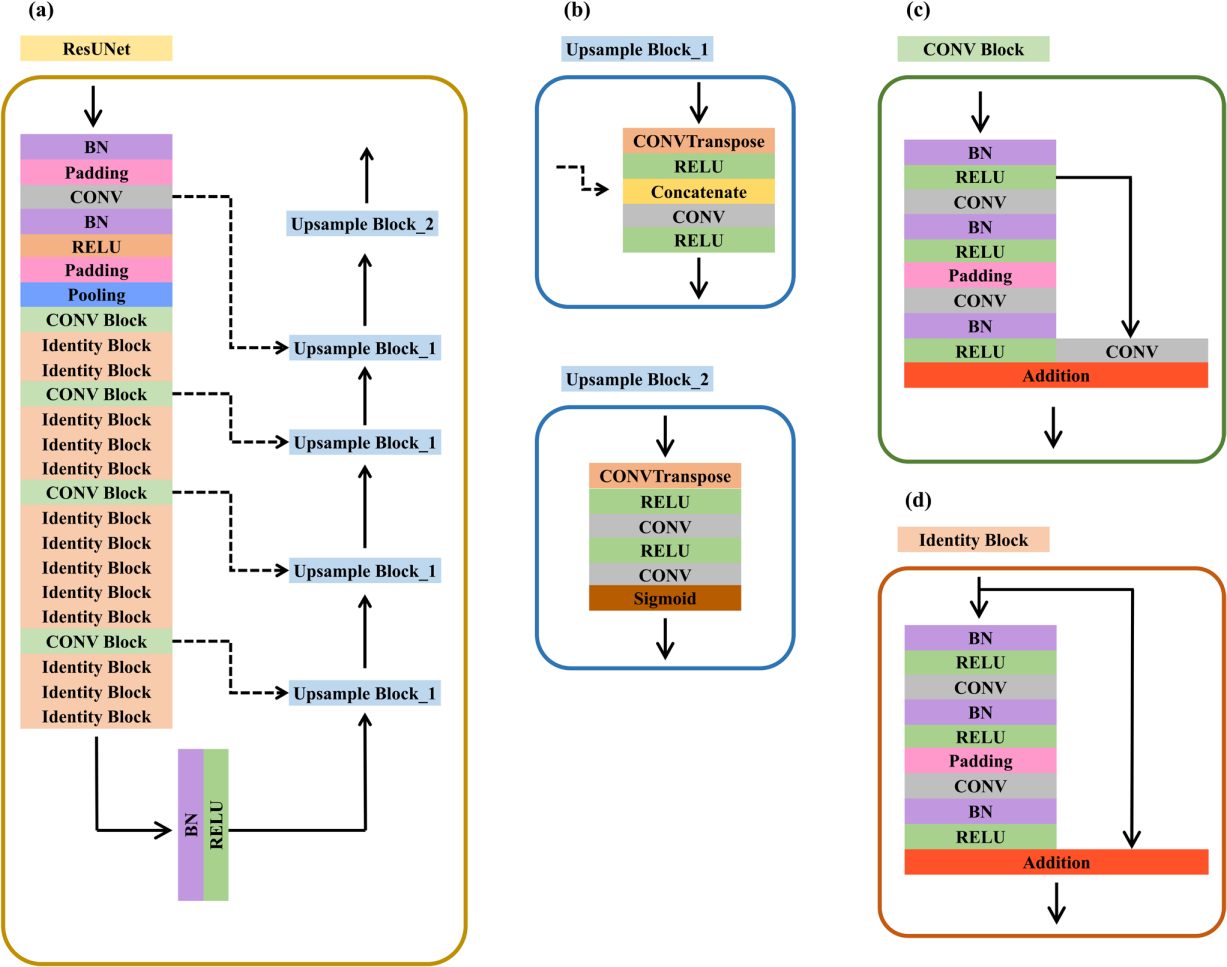
(a) The architecture of the model. (b) ResUNet Decoder reconstructs the reduced feature into a new image of the same size as the original image and adds concatenate to preserve the feature. (c) The residual blocks include the CONV block to allow the model to learn new features. (d) The Identity block maps the features of the previous level directly when the model does not learn new features so that the model will not degrade.

Figure 2 illustrates our deep‐learning model architecture based on a modified ResUNet framework, designed for image segmentation tasks for cervical ultrasound. Figure 2a presents the overall structure, with the main architecture being U‐Net, which includes both upsampling and downsampling processes. Starting with a ResNet backbone, the down‐sampling module first consists sequentially of Batch Normalization (BN), Padding, Convolution (CONV), Rectified Linear Unit (RELU) activations, padding, and pooling layers. Followed by a Pooling layer, the process continues through four stages, with each stage including two types of CONV blocks and Identity blocks. The first stage consists of a CONV block followed by two Identity blocks. The second and fourth stages each include a CONV block and three Identity blocks. The third stage comprises a CONV block and five Identity blocks. Finally, after the fourth stage, the architecture connects to BN layer followed by RELU activation layer.

Figure 2c describes the composition of the CONV block, which reduces the dimensionality of the feature maps. The layers in the CONV block sequentially arranged as BN, RELU, CONV, BN, RELU, Padding, Pooling, CONV, BN, and RELU layers. The output from the first RELU undergoes an additional CONV operation and is then added to the final RELU output to perform identity mapping, ensuring that the model can effectively learn the identity mapping. Figure 2d outlines the Identity block, which is mainly similar to the CONV block but does not reduce the dimensionality of the feature maps. In the Identity blocks, the output from the first RELU is directly added to the final RELU output for identity mapping. Identity blocks and CONV blocks enhance feature learning while preserving spatial information, which is crucial for effective feature extraction and maintaining the integrity of the features.

Figure 2b details the upsample blocks used in the model, critical for improving the resolution of the feature maps. The upsampling process consists of four Upsample Block_1 and Upsample Block_2 used to output the segmentation results. Upsample Block_1 includes a ConvTranspose layer for upsampling, followed by a RELU activation. The output is then concatenated with features from previous layers, followed by additional CONV and RELU layers. Regarding the concatenated method, the first Upsample Block_1 is connected to the output of the penultimate stage. By analogy, the second Upsample Block_1 is connected to the output of the third to last stage. The last Upsample Block_1 is connected to the first CONV layer of the downsampling process.

Upsample Block_2 starts similarly with a ConvTranspose layer and RELU activation but includes an extra CONV layer and ends with a Sigmoid activation function. This configuration helps refine the upsampled feature maps, ensuring higher quality outputs. Overall, the modified ResUNet architecture leverages residual learning and multiscale feature extraction, enhancing segmentation performance. The detailed inclusion of ConvTranspose layers for upsampling, along with the sequence of BN, RELU, and CONV layers, supports effective feature extraction and refinement. This approach ensures the model can capture a broader range of features, achieving superior generalization and robustness in image segmentation tasks.

### 2.6 Loss function

The loss function assesses the accuracy of a model's prediction relative to the ground truth, with lower values indicating greater accuracy. This study employs a loss function combining binary cross‐entropy and log dice loss. Binary cross‐entropy evaluates the performance of a model in predicting binary classification and is defined as Equation (i):

(i) $$\begin{array}{c} L_{\mathrm{BCE}}\left(x_{i,}{\tilde{x}}_{i}\right)=-\frac{1}{N}\sum_{i=1}^{N}x_{i}\log\left({\tilde{x}}_{i}\right)+\left(1-x_{i}\right)\log\left(1-{\tilde{x}}_{i}\right) \end{array}$$

$x_{i}$ is for the ground truth pixel values, ${\tilde{x}}_{i}$ is for the d predicted pixel values. When $x_{i}$ equals 1, and the predicted value ${\tilde{x}}_{i}$ approaches 1, the loss function's value should approach 0. Conversely, if the predicted value $x_{i}$ approaches 0 under the same condition, the loss function's value should become significantly high. The log dice loss is defined as Equation (ii):

(ii) $$\begin{array}{c} \begin{array}{c} L_{\text{LogDice}}\left(x_{i},{\tilde{x}}_{i}\right)=-\log\left(\frac{2\sum_{i=1}^{N}x_{i}{\tilde{x}}_{i}+\text{Smooth}}{\sum_{i=1}^{N}x_{i}+\sum_{i=1}^{N}{\tilde{x}}_{i}+\text{Smooth}}\right) \end{array} \end{array}$$

The log dice loss, derived from the Dice coefficient, measures the extent of overlap between the labeled and predicted images. The closer its value approaches 1, the greater the overlap observed.

### 2.7 Data augmentation

To enhance the outcomes of our model training, we implemented a comprehensive set of data augmentation techniques. Specifically, during the training process, we applied horizontal flipping to the ultrasound images to create mirror images, and we excised the noisy regions surrounding the images to reduce irrelevant data. In addition, we performed random rotations and scaling transformations to further diversify the data set. This augmentation process increased the number of images in the training data set from 1418 to 4254, while maintaining data integrity. By enhancing the data set's diversity, data augmentation facilitates the model's ability to learn a wider array of features and improves its generalization performance.

### 2.8 Training process

In our research, the data set underwent a strategic division aligned with conventional protocols for training machine‐learning models. Following the guidelines from the literature, the initial split between the training and testing data sets was made at a 90% and 10% ratio. From the training data set, a portion was further allocated to create a validation set. Consequently, the final distribution of the total data set was 75% for the training data set, 15% for the validation data set, and 10% for the testing data set. This allocation was meticulously planned to ensure a robust framework for the model's training and subsequent evaluation, essential for assessing its learning efficacy and generalization capability.

The model's training was conducted on a GeForce RTX 3090Ti GPU, with Intel i9‐12900K with 128 GB memory. This setup facilitated a batch size of 32 throughout an extensive training duration of 5000 epochs. During this period, we employed the Adam optimization algorithm in conjunction with a composite loss function. This loss function integrates Binary Cross‐Entropy and Dice Loss, a deliberate selection aimed at optimizing the model's proficiency in accurately detecting objects of varying sizes within the imaging data.^15^ The synergy of these loss functions is pivotal for augmenting the model's overall predictive accuracy.

Furthermore, our training protocol incorporated an Early Stopping mechanism. This methodological inclusion is designed to terminate the training process upon the detection that subsequent iterations fail to yield reductions in the loss metric observed on the training data set. The rationale behind integrating Early Stopping lies in enhancing the efficiency of the training process by precluding unnecessary computational expenditures.

The holistic approach employed in designing our training and testing methodologies was orchestrated with the objective of developing a model characterized by both high accuracy and operational efficiency. The culmination of these methodological efforts resulted in the formulation of a robust and reliable model, proficient in the precise identification of cervix locations within ultrasound imagery. This accomplishment signifies the model's substantial potential to make significant contributions to the domain of medical imaging, particularly in elevating the precision and effectiveness of diagnostic procedures.

### 2.9 Dice coeffieicent

We used the dice coefficient as a quantitative indicator for model evaluation, which is defined as Equation (iii):

(iii) $$\begin{array}{c} \begin{array}{c} \text{Dice}=\frac{2\sum_{i=1}^{N}x_{i}{\tilde{x}}_{i}}{\sum_{i=1}^{N}x_{i}+\sum_{i=1}^{N}{\tilde{x}}_{i}}=\frac{2\times \mathrm{TP}}{\left(\mathrm{TP}+\mathrm{FP}\right)+\left(\mathrm{TP}+\mathrm{FN}\right)} \end{array} \end{array}$$

TP (true positive) represents the image position of the endocervical canal, and the model predicts the endocervical canal at this position; FN (false negative) represents the image position of the endocervical canal, and the model does not predict the endocervical canal at this position; FP (false positive) represents the image position without an endocervical canal, and the model predicts the endocervical canal at this position.^15^

### 2.10 Structural Similarity Index

After completing the model training, we employed the Structural Similarity Index (SSIM) to assess the resemblance between the labeled and predicted images. SSIM is primarily used to compare the luminance, contrast, and structural attributes of two images and is given by Equation (iv):
(iv)

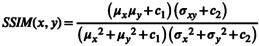


$\mu_{x}$ and $\mu_{y}$ represent the average of *x*, *y* respectively; $\sigma_{x}$ and $\sigma_{y}$ represent the standard deviation (SD) of *x*, *y* respectively; $\sigma_{\mathrm{xy}}$ represents the covariance of *x* and *y*; $c_{1}$ and $c_{2}$ are constants. SSIM values range from 0 to 1. A higher SSIM indicates lesser disparity between the two images. SSIM equals 1 when the images are identical.^16^ This choice is predicated on SSIM's ability to measure the perceptual quality of visual images, making it particularly suited for assessing the similarity between our model's outputs and actual images.

### 2.11 Measurement of CL

**FIGURE 3:**
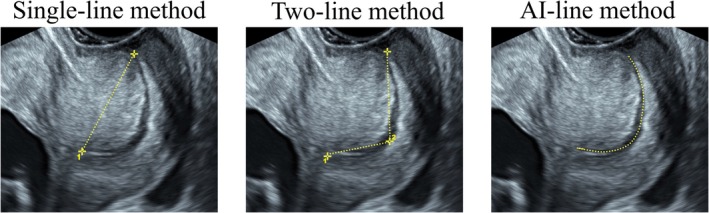
Transvaginal ultrasonographic CL measurements in the first trimester of pregnancy. (a) Single‐line method, as a straight line from the internal os to the external os. (b) Two‐line method, as the sum of two lines: One from the internal os to the greatest curvature, and the other from the end point of the first line to the external os. (c) AI‐line method, the length of the cervix by utilizing the scale between the internal and external cervical os (two ends of the glandular area around the endocervical canal) provided by tracing the endocervical canal to determine the actual length of the cervix. AI, artificial intelligence; CL, cervical length.

The CL was measured using three distinct methods. The first method followed the conventional approach as recommended by the FMF, which involves measuring the linear distance between the two ends of the glandular area around the endocervical canal (single‐line method) (Figure 3a). The second method involved the sum of two linear measurements: one from the internal os to the point of maximum cervical curvature, and the other from the end of the first line to the external os (two‐line method) (Figure 3b). The third method is to completely calculate the length of the cervix by utilizing the scale between the internal and external cervical os (two ends of the glandular area around the endocervical canal) provided by tracing the endocervical canal to determine the actual length of the cervix (AI‐line method) (Figure 3c).

### 2.12 Outcome measures

The primary outcome measure was sPTB at <37 weeks. The secondary outcome measures were sPTB at <32 weeks (early preterm) and 32 + 0 to 36 + 6 weeks (late preterm). sPTB included those with spontaneous onset of labor and those with preterm premature rupture of membrane (PPROM). Data on pregnancy outcomes were obtained from the maternity computerized records or the general medical practitioners of the participants and were recorded in our secure database. Missing outcomes (*n* = 46) were excluded from analysis. The obstetric records of all participants delivering at <37 weeks were reviewed to determine whether the PTB was medically indicated or spontaneous. The research team for the AI‐line method was blinded to outcome data.

### 2.13 Statistical analyses

Continuous variables were tested for normality using the Shapiro–Wilk test. Normally distributed variables were summarized as mean ± SD. CL measurements obtained from the three distinct methods were converted to multiples of the median (MoMs) and compared among groups with sPTB at <37 weeks, 32 to 36 + 6 weeks, and <32 weeks of gestation. Comparisons between the term delivery group and each spontaneous preterm group (<37 weeks, 32 to 36 weeks, and <32 weeks) were performed using Student *t* test. In addition, the Student *t* test was applied to assess differences between the AI‐line and single‐line methods, as well as between the AI‐line and two‐line methods. ne‐way analysis of variance was used to compare CL among the three outcome groups, followed by post hoc *t* tests for pairwise comparisons. Logistic regression models were constructed to assess the association between cervical‐length measurements and sPTB, adjusting for maternal age, BMI, parity, and prior PTB or cervical surgery. Statistical significance was defined as *P* < 0.05. All analyses were performed using Stata/SE version 14.0 (StataCorp). The study sample (*n* = 1664) provided >80% power to detect a 3‐mm difference in mean CL between the term and preterm groups.

### 2.14 Ethical approval

All participants provided written informed consent for participation in the original prospective study, which explicitly allowed future secondary use of anonymized ultrasound data for research. Ethical approval for this study was also given by the institutional review board (Joint Chinese University of Hong Kong—New Territories East Cluster Clinical Research Ethics Committee, Reference Number CRE‐2017.496). Before AI analysis, all images were de‐identified and re‐coded, with no patient identifiers remaining. Data were stored on secure institutional servers, accessible only to approved investigators under the ethical approval (NTUH 202207207RINC).

## 3 RESULTS

First‐trimester CL assessment was performed in a total of 1664 women with a singleton pregnancy. We excluded 77 cases (4.3%) attributed to missing outcome data (*n* = 46) and pregnancies resulting in miscarriage before 24 weeks of gestation (*n* = 18), stillbirth (*n* = 3), or termination (*n* = 10). Preterm birth at <37 weeks occurred in 126 (7.6%) cases and approximately two‐thirds of these were spontaneous (79 of 126; 4.7%), including 31 cases (1.8%) with spontaneous onset of labor and 48 cases (2.9%) with PPROM. Among these 79 cases, 68 (4.1%) and 11 (0.7%) women delivered spontaneously at 32 to 36 + 6 weeks and <32 weeks of gestation. Figure S1 illustrates the flow of participants in the study. Table 1 summarizes the baseline maternal and obstetric characteristics of the cohort.

**TABLE 1:** Maternal and pregnancy characteristics in the outcome groups.

Characteristic	Term delivery (*n* = 1585)	Spontaneous preterm birth		
		<37 weeks (*n* = 79)	32 to 36 weeks (*n* = 68)	<32 weeks (*n* = 11)
Maternal age, mean ± SD, years	32.8 ± 4.7	33.1 ± 4.9	33.0 ± 4.7	33.3 ± 5.2
Maternal height, median (IQR), cm	159 (156–163)	160 (155–164)	160 (156–163)	158 (154–161)
Maternal weight, median (IQR), kg	56.1 (50.0–62.8)	57.4 (51.9–64.0)	57.2 (52.3–63.6)	55.8 (49.5–66.2)
Body mass index, median (IQR), kg/m^2^	22.1 (20.0–24.2)	22.7 (21.1–24.9)	22.6 (21.0–24.8)	22.8 (19.9–25.4)
Cigarette smoker, *n* (%)	19 (1.2)	2 (2.5)	2 (2.9)	0
Gestational age at scan, median (IQR) weeks	12.6 (12.3–12.9)	12.5 (12.2–12.8)	12.5 (12.2–12.7)	12.4 (12.1–12.8)
Nulliparous, *n* (%)	928 (58.6)	49 (62.0)	42 (61.8)	7 (63.6)
Ethnicity				
Chinese, *n* (%)	1580 (99.7)	78 (98.7)	67 (98.5)	11 (100)
Non‐Chinese, *n* (%)	5 (0.3)	1 (1.3)	1 (1.5)	0
History of preterm birth, *n* (%)	52 (3.3)	8 (10.1)	7 (10.3)	1 (9.1)
Previous cervical surgery, *n* (%)	41 (2.6)	6 (7.6)	5 (7.4)	1 (9.1)

*Note*: Comparisons between term and preterm delivery groups were analyzed using *χ* ^2^ test or Fisher exact test for categorical variables and Mann–Whitney *U* test or *t* test for continuous variables.

Abbreviations: IQR, interquartile range; SD, standard deviation.

### 3.1 Model training and performance evaluation

**FIGURE 4:**
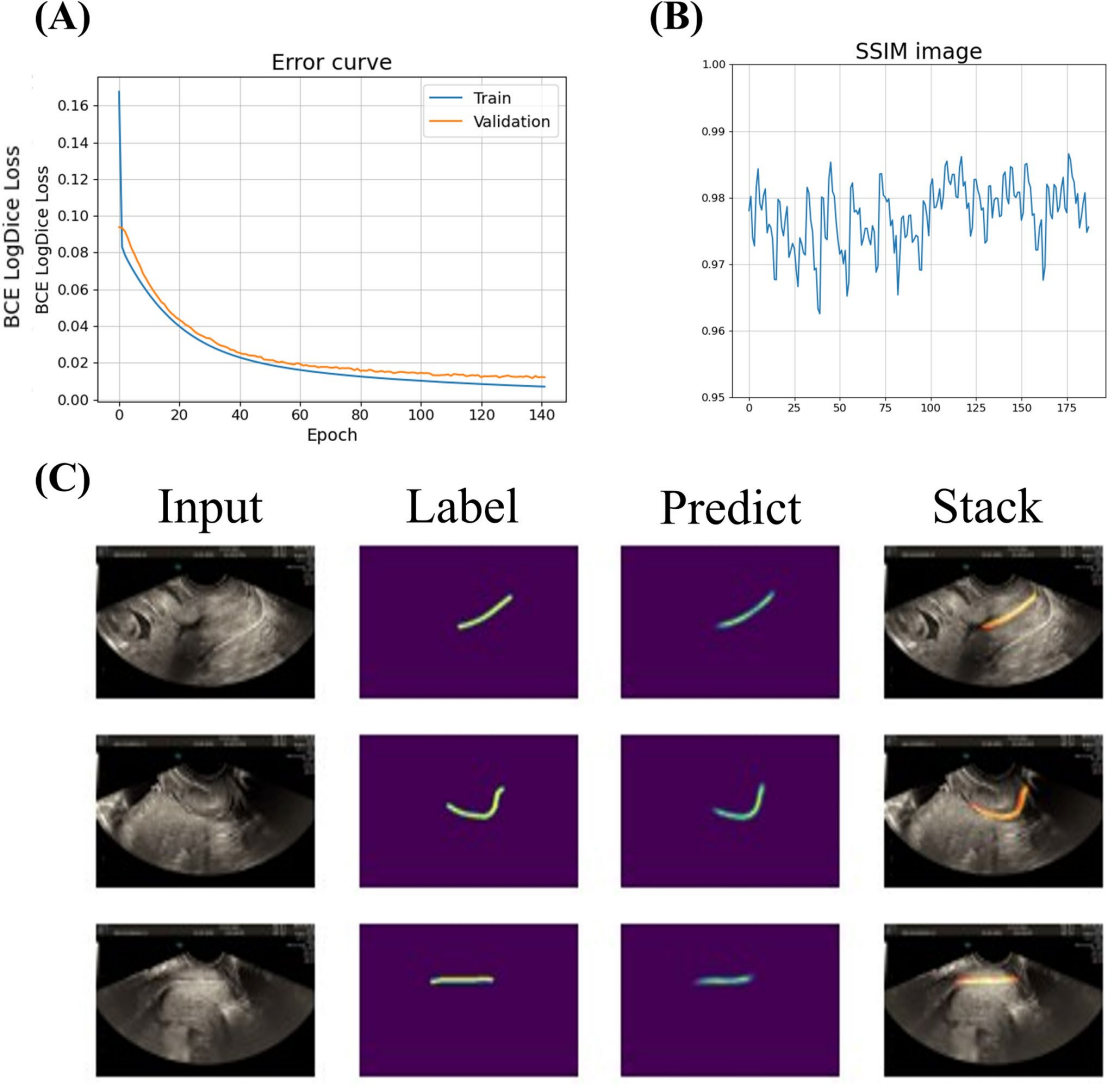
Training performance. (a) The error curve indicates that the training data set achieved a loss of approximately 0.0045 with an accuracy exceeding 99%, whereas the validation data set had a loss of approximately 0.0099 with an accuracy exceeding 99%. (b) Using the Structural Similarity Index (SSIM) as an indicator of label and prediction image similarity, most of the images have a similarity >0.96. (c) The deep‐learning model accurately predicts endocervical canal of the cervix in ultrasound images. Confirmation of training performance is demonstrated through the overlay of labeled and predicted images.

To elucidate the effectiveness of our training methodology and the model's predictive capabilities, an in‐depth analysis was conducted as outlined below. The meticulous training regimen of our model is shown in Figure 4, where BCE LogDice Loss was strategically selected as the loss function to critically evaluate the model's predictive accuracy. As demonstrated in Figure 4a, the model exhibited excellent performance in both the training and validation datasets, achieving a low loss of approximately 0.009 and an accuracy exceeding 99%.

### 3.2 Advancing image prediction accuracy with the SSIM and ResUNet framework

To advance the depth and precision of our evaluation concerning the congruence between algorithmically predicted images and their respective ground truth counterparts, we incorporated the SSIM as a critical evaluative metric. The analytical results, detailed in Figure 4b, revealed that an overwhelming majority of SSIM scores exceeded a threshold of 0.97. Such high SSIM values underscore marked fidelity between the predicted ultrasound images by our model and the authentic ultrasound images, highlighting the model's effectiveness in capturing the intricate details necessary for accurate image prediction.

### 3.3 Comparative analysis and clinical implications

To rigorously substantiate the model's predictive accuracy, we performed a comparative analysis involving the superimposition of the labeled ground truth images over the model's predictions. The ensuing visual comparison, as illustrated in Figure 4c, displays a substantial overlay between the labeled and the algorithmically predicted images. This significant overlap is indicative of the model's high level of accuracy in localizing the cervix within ultrasound scans, providing a compelling evidence base for the model's applicability in enhancing diagnostic imaging protocols. This comprehensive approach not only affirms the model's precision but also underscores its potential to contribute significantly to the field of medical imaging, particularly in the context of improving the accuracy and reliability of cervical assessments in ultrasound studies.

### 3.4 Comparative efficacy of the AI‐line method in predicting sPTB

**FIGURE 5:**
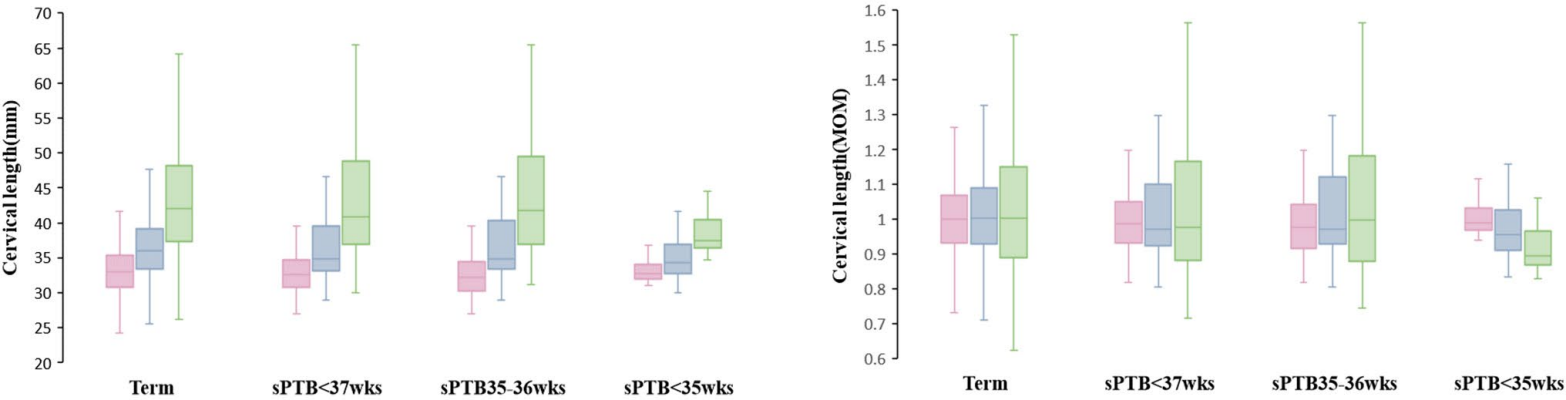
A box whisker plot was employed to visualize the distribution of (a) CLand (b) its MoM values between the sPTB <37 weeks, sPTB at 32 to 37 weeks, and sPTB <32 weeks groups. The plot includes data from the single‐line method (shown in pink), the two‐line method (shown in blue), and the AI‐line method (shown in green). CL, cervical length; MoM, multiple of the median; sPTB, spontaneous preterm birth.

The evaluation of the AI‐line method for CL measurement in the first trimester highlights its potential in predicting sPTB. In Table 2, the AI‐line approach to assessing CL during the first trimester showed a significant decrease in CL among women who experienced sPTB before <37 weeks of gestation (38.8 ± 6.5 mm) compared with those who delivered at term (43.3 ± 9.2 mm) in Table 2. When analyzing CL variations among different cohorts of sPTB, the AI‐line method consistently indicated a marked reduction in CL for deliveries between 32 to 36 + 6 weeks of gestation (38.9 ± 6.9 mm) and before 32 weeks (37.3 **±** 1.9 mm), compared to term birth. Notably, this specific pattern of measurement was not observed with the use of traditional single‐line or two‐line methods. Furthermore, the AI‐line method's measurements exhibited significant statistical differences, especially when comparing groups of sPTB with term birth, underscoring the method's distinctiveness and reliability over conventional methods. The analysis also revealed uniform trends in the MoM values for CL, as calculated by these three distinct approaches, further validating the AI‐line method's consistency and accuracy in Table 2 and Figure 5.

**TABLE 2:** Comparison of CL measured in single‐line, two‐line, and AI‐line methods in different outcome groups.

	Term birth ≥37 weeks	Spontaneous preterm birth		
		<37 weeks	32 to 36 weeks	<32 weeks
No. (%)	1585 (95.3)	79 (4.7)	68 (86.1)	11 (13.9)
CL, mm				
Single‐line method	33.6 ± 3.6	33.3 ± 3.3	33.2 ± 3.5	33.4 ± 1.2
Two‐line method	36.9 ± 4.6	36.4 ± 4.3	36.6 ± 4.5	35.4 ± 2.8
AI‐line method	43.3 ± 9.2	38.8 ± 6.5 (*P* < 0.0001)*	38.99 ± 6.9 (*P* < 0.0001)**	37.3 ± 1.9 (*P* = 0.02)***
CL, MoM				
Single‐line method	1.01 ± 0.1	0.996 ± 0.09	0.995 ± 0.1	0.999 ± 0.04
Two‐line method	1.02 ± 0.1	1.004 ± 0.1	1.008 ± 0.1	0.98 ± 0.08
AI‐line method	1.05 ± 0.2	0.941 ± 0.2 (*P* < 0.0001)*	0.946 ± 0.2 (*P* = 0001)**	0.906 ± 0.05 (*P* = 0.02)***

*Note*: Data are given as median (interquartile range).

Abbreviations: AI, artificial intelligence; CL, cervical length; MoM, multiple of the median.

* *P* < 0.05 for comparisons between term and spontaneous preterm birth <37 weeks.

** *P* < 0.05 for comparisons between term and spontaneous preterm birth at 32 to 36 weeks.

*** *P* < 0.05 for comparisons between term and spontaneous preterm birth <32 weeks.

### 3.5 Analyzing the effectiveness of measurement methods in predicting sPTB

**FIGURE 6:**
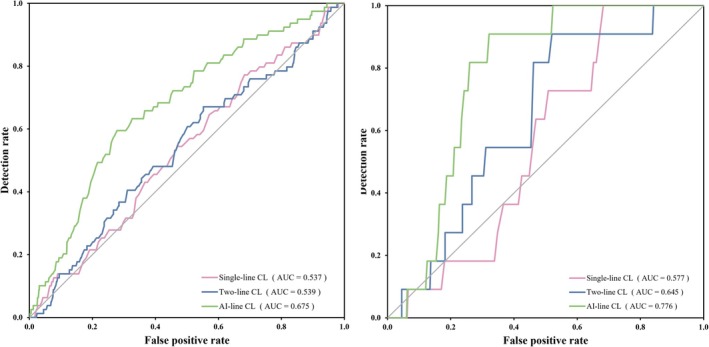
Receiver operating characteristics curve for the prediction of sPTB by CL. (a) sPTB <37 weeks of gestation and (b) sPTB <32 weeks of gestation. The receiver operating characteristics curves were plotted for each of the measurement methods: The single‐line method (pink line), two‐line method (blue line), and AI‐line method (green line). AI, artificial intelligence; AUC, area under the curve; CL, cervical length; sPTB, spontaneous preterm birth.

Our study focuses on the predictive capability of different measurement methods for sPTB, emphasizing the importance of CL as a critical factor. The area under the receiver operating curve (AUROC) values are shown in Table 3 and Figure 6. In the prediction of early sPTB <37 weeks (Figure 6a), the AUROC of the CL measured by the AI‐line method (0.676 [95% CI, 0.616–0.735], *P* < 0.05) was significantly higher than the single‐line (0.537; 95% CI, 0.474–0.600) and two‐line (0.54; 95% CI, 0.473–0.660) methods. For the prediction of sPTB at <32 weeks, as depicted in Figure 6b, this trend was maintained with the AI‐line method achieving an AUROC of 0.777 (95% CI, 0.703–0.85), markedly higher than that of the single‐line (0.577; 95% CI, 0.46–0.693) and two‐line (0.646; 95% CI, 0.514–0.778) methods.

**TABLE 3:** Performance of screening for spontaneous preterm birth with CL measured in single‐line, two‐line, and AI‐line methods.

	Spontaneous preterm birth		
	<37 weeks	32 to 36 weeks	<32 weeks
AUROC, %			
Single‐line method	53.7 (47.4–60)	53.1 (46–60.1)	57.7 (46–69.3)
Two‐line method	54 (47.3–6.6)	52.3 (45–59.6)	64.6 (51.4–77.8)
AI‐line method	67.6 (61.6–73.5) *(*P* < 0.0001) ^†^(*P* < 0.0001)	65.9 (59.2–72.6) *(*P* < 0.0001) ^†^(*P* < 0.0001)	77.7 (70.3–85) *(*P* = 0.0002) ^†^(*P* = 0.0014)

Abbreviations: AI, artificial intelligence; AUROC, area under the receiver operating characteristic; CL, cervical length.

* *P* < 0.05 for comparisons between AI‐line and single‐line method.

^†^ *P* < 0.05 for comparisons between AI‐line and two‐line method.

## 4 DISCUSSION

### 4.1 Principle findings

The findings of this investigation revealed that, first, the conventional approach for the measurement of CL with the single‐line and two‐line methods in the first trimester of pregnancy underestimates the actual CL, Second, the CL measured using the AI‐line method exhibits superior efficacy in predicting sPTB compared with CL measurements derived from the single‐line and two‐line methods. The utilization of the AI‐line method facilitates a more accurate estimation of the actual endocervical length.

### 4.2 Results

In consideration of the curved cervix in the first trimester of pregnancy, we have evaluated the potential value of the CL measured by the AI‐line method, which accounts for the curvature of the cervix, for the prediction of sPTB, and, to our knoweledge, this has not been previously explored. Our findings underscore the superiority of the AI‐line method in the early prediction of sPTB. The AUROC analysis corroborated the enhanced performance of the AI‐line method over traditional single‐line and two‐line approaches in the first‐trimester CL measurement for predicting sPTB before both <37 weeks and <32 weeks of gestation, as evidenced by a larger area under the curve. This analytical outcome firmly supports the adoption of the AI‐line method for transvaginal ultrasonographic assessment of CL as an effective screening tool. It demonstrates considerable promise for the early detection of women at risk of sPTB, thereby facilitating timely intervention and management in the initial stages of pregnancy.

### 4.3 Clinical and research implications

No prior research, to our knowledge, has examined a large data set using AI to automatically label ultrasound images of the sagittal plane of the cervix for data analysis and to explore the association of sPTB. Retzke et al.^17^ compared the single‐line, two‐line, and tracing methods of CL measurement, which relied on manual techniques using transvaginal ultrasound in the first trimester. The study suggested that the two‐line and tracing methods provided more accurate measurements, particularly for curved cervices. Our study used AI to label “the tracing method” to determine the length of the cervix, which presented a more advanced and potentially transformative approach to measuring CL. Furthermore, the manual tracing method does not provide specific predictive values for PTB. Our AI‐line method addressed the challenges of manual measurements by providing a more accurate prediction of PTB.

A recent systematic review and meta‐analysis^18^ have also stressed that women with sPTB have significantly shorter transvaginal CL before 16 weeks of gestation compared with those who deliver at term. The study has also indicated that the linear method and the 2‐line method are acceptable techniques for measuring transvaginal C, but standardized protocols and further research to refine predictive cutoffs and techniques are needed. Ultrasound images represent calibrated visual interpretation based on the operator's knowledge and perception, which necessitates a significant learning curve for effective ultrasound use and interpretation. Performing ultrasonography, especially the transvaginal ultrasound, requires a high level of training that may take years to develop. Our study has provided a technological approach using a modified ResUNet framework and AI‐empowered tools, which could eventually help nonradiology‐trained clinicians in measuring the CL.

### 4.4 Strengths and limitations

The AI‐line method, which integrates UNet and ResNet architectures into a ResUNet‐based model, is a novel approach that could predict sPTB more reliably compared with both the two‐line and single‐line methods. One limitation of our study is that it was conducted at a single center, which may limit the generalizability of the findings. Multicenter validation would strengthen the evidence for clinical implementation. The other limitation of our study includes its retrospective design and the small number of events, with only 11 cases of sPTB at <32 weeks, but these cases were sufficient for evaluating a single marker for the prediction of sPTB. Despite the relatively small sample size used for training the ResUNet‐based model, we obtained highly significant results.

## 5 CONCLUSION

The findings of the current study highlight the potential of AI to transform the screening process for sPTB. The AI‐line method, which integrates UNet and ResNet architectures into a ResUNet‐based model, has demonstrated significant advantages over traditional single‐line and two‐line measurement methods. The AI‐line method not only provides a more accurate measurement of CL during the first trimester of pregnancy but also exhibits superior predictive capabilities for assessing the risk of sPTB.

Moreover, the study results underscore the potential for incorporating AI technologies in prenatal care, emphasizing the need for prenatal imaging as a part of routine clinical care. While the research has limitations, the promising results indicate a significant opportunity to enhance clinical practices. As AI models become more advanced and data sets expand, the accuracy and reliability of sPTB prediction are expected to improve, making AI an essential tool in the future of obstetric care.

## AUTHOR CONTRIBUTIONS

Yi‐yun Tai: Data curation, writing–original draft preparation. Liona C. Poon: Supervision. Chi‐Hua Yu: Conceptualization, methodology, software. Bor‐Yann Tseng: Software, validation. Zhu‐Han Yang: Data curation, software.

## FUNDING INFORMATION

This study was conducted under the National Science and Technology Council (MOST 112WFA0110012) and was supported in part by Higher Education Sprout Project, Ministry of Education to the Headquarters of University Advancement at National Cheng Kung University (NCKU). This study was also supported by the Hong Kong Innovation and Technology Support Program ITF funding (ITF No: ITS/252/17FX) and a startup grant from the Faculty of Medicine, The Chinese University of Hong Kong. We also thank Ching Man Mak, Maggie S.M. Mak, Angela S.T. Tai, Tracy C.Y. Ma, Kimberley Zhang, and Katrina M.H. Ng in assisting with patient recruitment and ultrasound assessment; and the members of nurses, midwives, and physicians at the Prince of Wales Hospital in facilitating the performance of this study.

## CONFLICT OF INTEREST STATEMENT

The authors declare no competing interests regarding this article.

## Supporting information


**Figure S1.** The flow of participants in the study. TOP, termination of pregnancy; sPTB, spontaneous preterm birth; PPROM, preterm prelabor rupture of membranes.

## Data Availability

Research data are not shared.
