# Supplementary material for: Artificial intelligence application in the prediction of spontaneous preterm birth by cervical length in the first trimester of pregnancy: Comparison of three measurement methods
Source: Int J Gynaecol Obstet. 2026 Jan 9;173(3):1421–32. doi: 10.1002/ijgo.70744 (PMC13173632; doi:10.1002/ijgo.70744)
Supplement: Supplementary file 1 — Figure S1. The flow of participants in the study. TOP, termination of pregnancy; sPTB, spontaneous preterm birth; PPROM, preterm prelabor rupture of membranes. [file IJGO-173-1421-s001.docx]

**Supplement Figure**

**
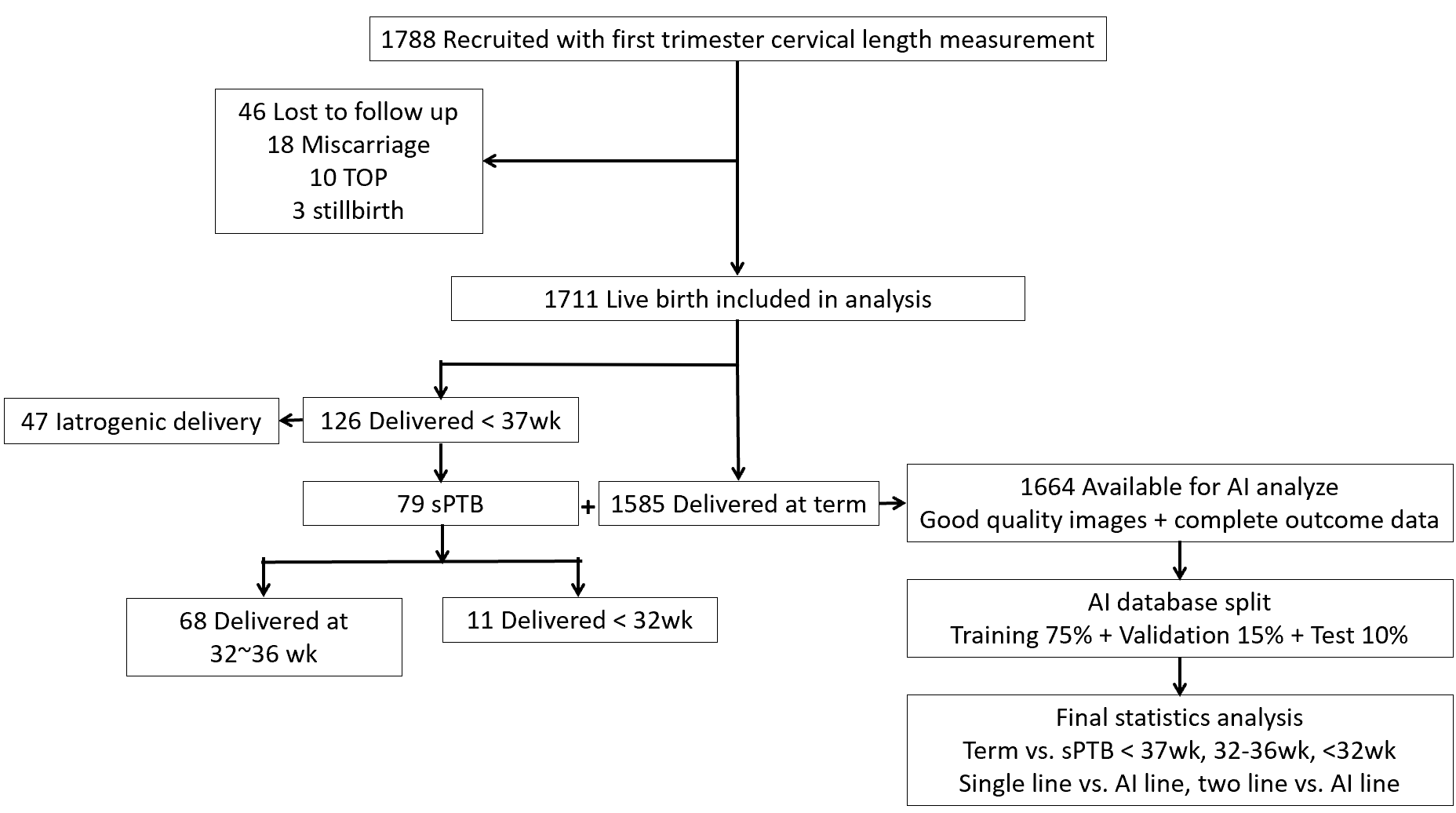
**

**Figure S1 The flow of participants in the study.**

TOP, termination of pregnancy; sPTB, spontaneous preterm birth; PPROM, preterm prelabor rupture of membranes
